# Encephalopathy and Encephalitis Associated with Cerebrospinal Fluid Cytokine Alterations and Coronavirus Disease, Atlanta, Georgia, USA, 2020

**DOI:** 10.3201/eid2609.202122

**Published:** 2020-09

**Authors:** Karima Benameur, Ankita Agarwal, Sara C. Auld, Matthew P. Butters, Andrew S. Webster, Tugba Ozturk, J. Christina Howell, Leda C. Bassit, Alvaro Velasquez, Raymond F. Schinazi, Mark E. Mullins, William T. Hu

**Affiliations:** Emory University School of Medicine, Atlanta, Georgia, USA

**Keywords:** coronavirus disease, COVID-19, severe acute respiratory syndrome coronavirus 2, SARS-CoV-2, coronaviruses, viruses, encephalopathy, encephalitis, meningitis/encephalitis, cerebrospinal fluid, CSF, cytokines, serologic analysis, respiratory infections, zoonoses, Atlanta, Georgia, United States

## Abstract

There are few detailed investigations of neurologic complications in severe acute respiratory syndrome coronavirus 2 infection. We describe 3 patients with laboratory-confirmed coronavirus disease who had encephalopathy and encephalitis develop. Neuroimaging showed nonenhancing unilateral, bilateral, and midline changes not readily attributable to vascular causes. All 3 patients had increased cerebrospinal fluid (CSF) levels of anti-S1 IgM. One patient who died also had increased levels of anti-envelope protein IgM. CSF analysis also showed markedly increased levels of interleukin (IL)-6, IL-8, and IL-10, but severe acute respiratory syndrome coronavirus 2 was not identified in any CSF sample. These changes provide evidence of CSF periinfectious/postinfectious inflammatory changes during coronavirus disease with neurologic complications.

The pandemic caused by severe acute respiratory syndrome coronavirus 2 (SARS-CoV-2) has led to >1.5 million infections in the United States (30% of global cases) and >90,000 deaths as of May 20, 2020 (*1*). Coronavirus disease (COVID-19, the clinical syndrome associated with SARS-Cov-2) is most commonly characterized by respiratory illness and viral pneumonia with fever, cough, and shortness of breath, and progression to acute respiratory distress syndrome in severe cases (*2*).

Although neurologic complications have been noted in previous human coronavirus infections (*3*–*5*), there are few in-depth investigations for neurologic syndromes associated with SARS-CoV-2 infection (*6*). This deficiency can result from the need to reduce unnecessary staff exposure and difficulties in establishing preillness neurologic status without regular family visitors. It is known that neurons and glia express the putative SARS-CoV-2 receptor angiotensin converting enzyme 2 (*7*), and that the related coronavirus SARS-CoV (responsible for the 2003 SARS outbreak) can inoculate the mouse olfactory bulb (*8*). If SARS-CoV-2 can enter the central nervous system (CNS) directly or through hematogenous spread, cerebrospinal fluid (CSF) changes, including viral RNA, IgM, or cytokine levels, might support CNS infection as a cause for neurologic symptoms. We report clinical, blood, neuroimaging, and CSF findings for 3 patients with laboratory-confirmed COVID-19 and a range of neurologic outcomes (neuro-COVID). We also show the presence of SARS-CoV-2 antibodies in the blood and CSF of these patients, consistent with CNS penetration of disease.

## Methods

We describe the clinical, laboratory and radiologic findings for 3 patients with respiratory failure and neurologic complications caused by COVID-19. This case series was reviewed and exempted from Emory Institutional Review Board approval. Medical records were reviewed by 4 of the coauthors (K.B., A.A., M.E.M., and W.T.H.).

### CSF Serologic Analysis, Cytokines, and Molecular Testing

We assessed CSF IgM by using an in-house ELISA against SARS-CoV-2 S1 or envelope (E) protein. This ELISA was modified from an in-house blood-based ELISA with 90% sensitivity and 89% specificity for confirmed COVID-19 against 78 pre-2020 controls. CSF was serially diluted from 1:2 to 1:16, and CSF from 1 case-patient who had HIV infection (hospitalized during March 2020) and from 3 pre-2020 healthy subjects (*9*) were included for comparison. We measured levels of plasma IgG against the receptor-binding domain of S1 by using a commercial ELISA (GenScript, https://www.genscript.com) at a 1:16 dilution.

We analyzed CSF inflammatory proteins (MilliporeSigma, https://www.emdmillipore.com) by using a Luminex-200 platform and a modified manufacturer’s protocol as described (*9*). These proteins include interleukin (IL)-1α, IL-1β, IL-2, IL-4, IL-6, IL-7, IL-8, IL-9, IL-10, IL12-p40, IL12-p70, interferon-gamma–induced protein 10 (IP-10), monocyte chemoattractant protein 1 (MCP-1/CCL2), macrophage-derived chemokine (MDC/CCL22), fractalkine (CX3CL1), and tumor necrosis factor α (TNF-α).

We performed molecular testing for SARS-CoV-2 by using real-time quantitative reverse transcription PCR (qRT-PCR). We extracted total nucleic acid from 120 µL of CSF from each person by using the EZ1 Virus Mini Kit version 2.0 and the EZ1 Advanced XL Instrument (QIAGEN, https://www.qiagen.com) after lysis with AVL lysis buffer (QIAGEN). We performed a 1-step qRT-PCR by using 2019-nCoV_N1 or 2019-nCoV_N2 combined Primer/Probe Mix (Integrated DNA Technologies, Inc., https://www.idtdna.com) in a Roche LightCycler 480 II, (https://lifescience.roche.com), an endogenous control, and an in vitro transcribed full-length RNA of known titer (Integrated DNA Technologies, Inc.) as a positive control. We followed the same procedure for influenza A virus except using a primer/probe mixture (*10*) and a mitochondrial cytochrome oxidase subunit 2 DNA endogenous control (*11*). We tested all samples in duplicate.

## Results

### Clinical, Radiologic, and Laboratory Assessment

Patient 1, a 31-year-old African-American woman who had sickle cell disease (SCD) and was receiving dabigatran for a recent pulmonary embolus, came to a community hospital after 5 days of progressive dyspnea. An initial chest radiograph showed a right lower lobe infiltrate, and she was given a blood transfusion and antimicrobial drugs for presumed SCD crisis and pneumonia. Her breathing became more labored, and a repeat chest radiograph showed worsening bilateral infiltrates. A nasopharyngeal swab specimen was positive for SARS-CoV-2 and influenza A virus (negative for influenza B virus). She was empirically given hydroxychloroquine (400 mg daily) and peramivir (100 mg daily), but acute kidney injury and progressive hypoxemic respiratory failure developed. She was intubated and transferred to our institution on day 11. Her paralysis and sedation were discontinued on day 13 after improved oxygenation, but she remained comatose with absent brainstem reflexes on day 15.

**Figure 1:**
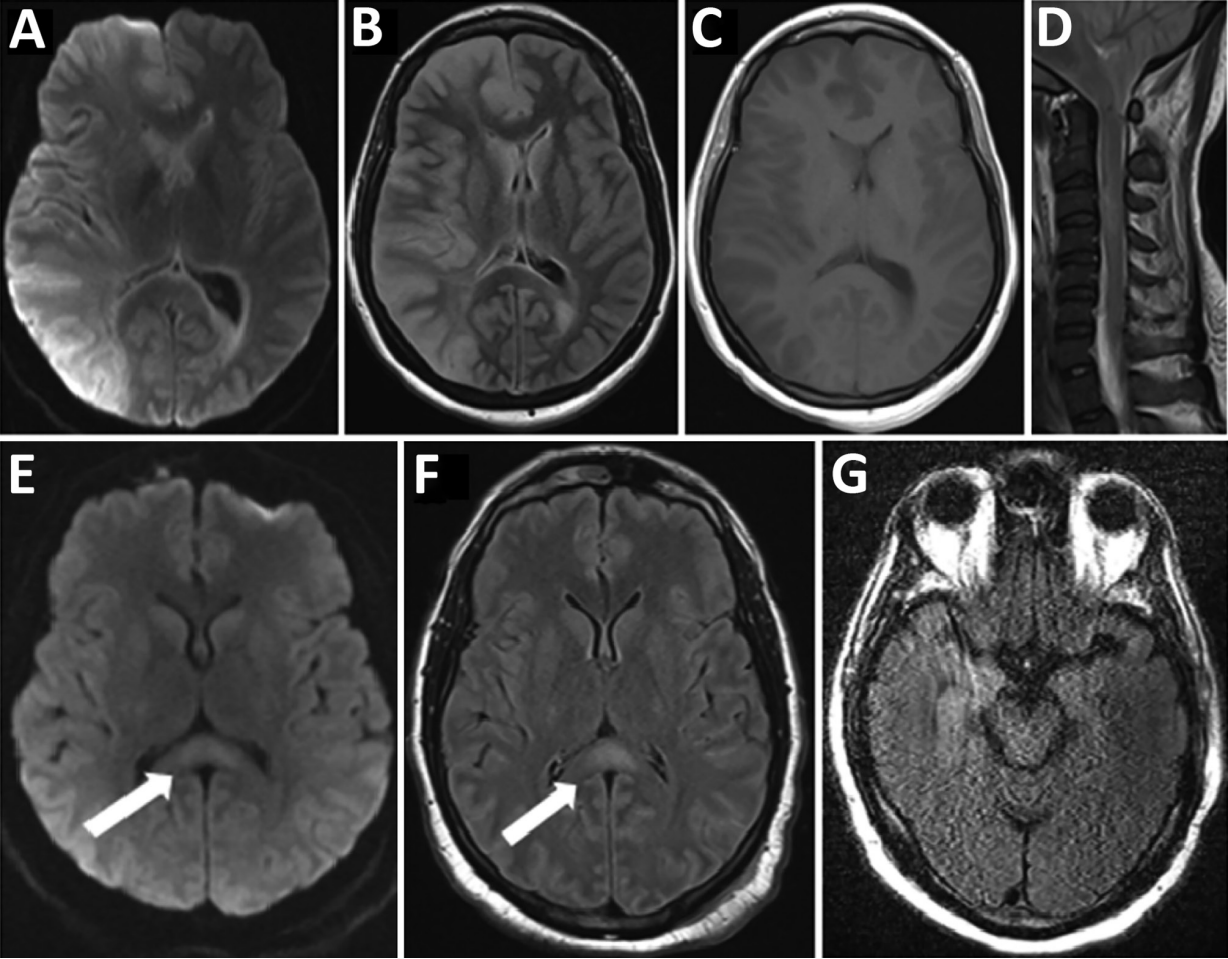
Magnetic resonance imaging findings for 3 patients with coronavirus disease who had neurologic complications, Atlanta, Georgia, USA, 2020. A–D) Patient 1 had right cerebral hemispheric restricted diffusion (diffusion weighted imaging in panel A) and cerebral edema (fluid-attenuated inversion recovery [FLAIR] in panel B) affecting gray matter and deep gray nuclei, without enhancement (panel C), and spinal edema (panel D). E, F) Patient 2 had a splenium lesion (diffusion weighted imaging in panel E and FLAIR recovery in panel F that was nonenhancing). Arrows indicate lesions in the splenium. G) Patient 3 had an equivocal fluid-attenuated inversion recovery FLAIR abnormality in the right temporal lobe.

Brain magnetic resonance imaging (MRI) showed nonenhancing cerebral edema and diffusion weighted imaging abnormalities predominantly involving the right cerebral hemisphere, as well as brain herniation (Figure 1). An occlusive thrombus was identified in the right internal carotid artery, and edema was also identified in the cervical spinal cord. The overall appearance was most consistent with encephalitis and myelitis, with superimposed hypoxic ischemic changes. CSF showed high opening pressure of 30 cm of water, 115 nucleated cells/mL, 7,374 erythrocytes/mL, an increased protein level (>200 mg/dL), and a glucose level within a standard range (Table). Her nucleated cell count remained strongly increased even after correction for the traumatic tap (≈1 nucleated cell/700 erythrocytes). Given a grave prognosis, the family withdrew life-sustaining care and the patient died on day 16.

**Table:** Characteristics of 3 patients with coronavirus disease and neurologic complications, Atlanta, Georgia, USA, 2020*

Characteristic	Patient 1	Patient 2	Patient 3
Neurologic findings			
Encephalopathy	Coma	Moderate	Mild
Brainstem reflexes affected	All	Corneal gag	Oculocephalic
Myoclonus	None	Arms	All limbs
Withdrawal to pain	Absent	Absent	Absent
CSF findings, reference value			
Appearance, clear	Cloudy	Clear	Clear
Opening pressure, 10–20 cm H_2_O	30	48	12
Nucleated cells			
Total, 0–5/μL	115	1	0
% Neutrophils, 0%–6%	51	75	0
% Lymphocytes, 40%–80%	10	25	0
% Macrophages 15%–45%	39	0	0
Erythrocytes, 0/μL	3,426	29	7
Glucose, 40–70, mg/dL	40	111	88
Protein, 15–45, mg/dL	>200	37	21

*CSF, cerebrospinal fluid.

Patient 2, a 34-year-old African-American man who had hypertension, showed development of fever, shortness of breath, and cough. Computed tomography of the chest showed bilateral, diffuse ground glass infiltrates. A nasopharyngeal swab specimen obtained on day 1 showed SARS-CoV-2. He was given a 6-day course of hydroxychloroquine, but hypoxic respiratory failure developed, which required intubation, followed by encephalopathy with myoclonus on day 9. His neurologic examination showed profound encephalopathy, absent corneal and gag reflexes, multifocal myoclonus involving both arms, and absent withdrawal to painful stimuli. Electroencephalography showed diffuse slowing with a suggestion that the myoclonus was seizure-related. Brain MRI on day 15 showed a nonenhancing hyperintense lesion within the splenium of the corpus callosum on fluid-attenuated inversion recovery and diffusion weighted imaging sequences (Figure 1). CSF showed high opening pressure of 48 cm H_2_O, no pleocytosis, 27 erythrocytes/mL, a mildly increased protein level, and glucose level within the reference range.

Patient 3, a 64-year-old African-American man who had hypertension, showed development of cough, dyspnea, and fever with multifocal, patchy, ground glass opacities on chest computed tomography and a nasopharyngeal swab specimen positive for SARS-CoV-2. His symptoms progressed to hypoxic respiratory failure requiring intubation, and his multifocal myoclonus began soon after starting to take hydroxychloroquine. His neurologic examination showed profound encephalopathy, absent oculocephalic reflex, multifocal myoclonus affecting bilateral arms and legs, absent withdrawal to pain, and diminished deep tendon reflexes. The resolution of his myoclonus coincided with fentanyl cessation, but it is not clear that the 2 symptoms were related. A motion-degraded brain MRI showed an equivocal nonenhancing area of fluid-attenuated inversion recovery abnormality in the right temporal lobe. CSF obtained on hospital day 11 showed a normal opening pressure; levels of nucleated cells, erythrocytes, and protein within reference ranges; and an increased glucose level (Table). His mentation began to improve on day 13, and he was subsequently discharged without major neurologic sequelae.

### Serologic Analysis of Plasma and CSF

Plasma anti-S1 receptor-binding domain IgG levels were increased for all 3 patients, consistent with severe COVID-19 (T. Ozturk et al., unpub. data). An indirect ELISA for plasma showed an increased level of anti-S1 IgM for patients 1 (1:512) and 2 (1:256), a highly increased level of anti-S1 IgM for patient 3 (1:2,048); an increased level of anti-E IgM for patients 1 and 2 (1:128), and a standard level of anti-E IgM for patient 3.

**Figure 2:**
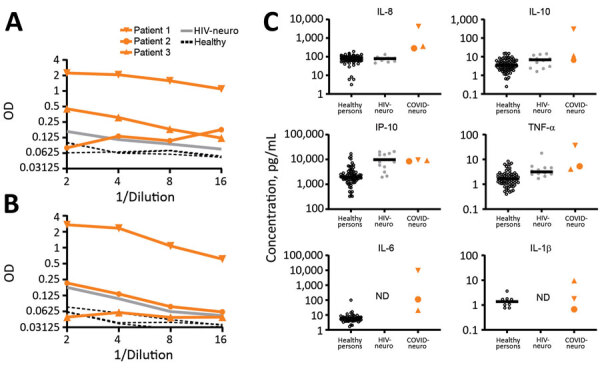
Cerebrospinal fluid (A and B) and inflammatory protein (C) analyses for patients with coronavirus disease and neurologic complications, Atlanta, Georgia, USA, 2020. Compared with healthy controls and patients who had HIV-associated neurocognitive disorder, CSF levels of anti-S1 IgM were high in patient 1, and moderately high in patients 2 and 3. In contrast, levels of CSF anti-E IgM were high only for patient 1 and within references ranges for patients 2 and 3. CSF inflammatory analysis showed increased levels of IL-8 and IL-10 more unique to neuro-COVID, and increased levels of IP-10 and TNF-α in neuro-COVID and HIV-neuro. Circles indicate patients whose interleukin levels were tested and used as controls (healthy, HIV). Horizontal bars indicate average values. COVID-neuro, coronavirus disease–associated neurologic complications; HIV-neuro, HIV-associated neurocognitive disorder; IL, interleukin; IP, interferon-γ–induced protein; OD, optical density, ND, not determined; neuro-COVID, neurologic complications associated with coronavirus disease; TNF, tumor necrosis factor.

An indirect ELISA for CSF showed markedly increased levels of IgM for SARS-CoV-2 S1 (Figure 2, panel A) and E (Figure 2, panel B) proteins for the most severely ill patient 1, and mildly elevated levels of IgM for S1 only for patients 2 and 3. The number of CSF erythrocytes in patient 1 suggested plasma contamination at an approximate dilution of 1:1,000, which still placed these CSF IgM levels higher than those for patients 2 and 3.

### Inflammatory Protein Analysis for CSF

CSF from patients 1 and 3 underwent detailed inflammatory protein profiling as described (*9*,*12**,**13*). When we compared historical and present control subjects who had normal cognition (no viral illness) (*13*), we found that patients with COVID-19 and neurologic symptoms had increased CSF levels of IL-6, IL-8, IL-10, IP-10, and TNF-α (Figure 2, panel C). Levels of IL-8, IL-10, IP-10, and TNF-α were also available for subjects who had HIV-associated neurocognitive disorders (*12*). Increased levels of IL-8 and IL-10 appeared to be unique for neurologic complications of SARS-CoV-2, and increased levels of IP-10 and TNF-α were common features between neurologic complications of SARS-CoV-2 and HIV.

### Viral Analysis of CSF

We used a real-time RT-PCR to test for SARS-CoV-2 and influenza A virus (tested because patient 1 showed a co-infection). Results were negative for all patients.

## Discussion

We report 3 patients who had severe COVID-19 and showed development of various neurologic symptoms and findings in a US hospital. All patients had more severe symptoms affecting cortical and brainstem functions at the peak of their neurologic illnesses than a recent series of 7 case-patients with milder illness in France (*6*). All 3 patients were also co-incidentally given a short course of empiric hydroxychloroquine, although there was no temporal correlation between the medication and their neurologic manifestation. Similar to the case-series in France, we did not isolate SARS-CoV-2 RNA from CSF, although such viral RNA has been inconsistently identified in other cases (*14*). However, increased levels of CSF anti-S1 IgM and altered levels of CSF cytokines are consistent with direct CNS involvement by SARS-CoV-2. Because MRI changes seen in these patients could be caused by hypercoagulability (*15*) or metabolic encephalopathy (*16*), we propose that CSF investigation can improve the distinction between neurologic involvement of SARS-CoV-2 (or neuro-COVID) and neurologic symptoms caused by other COVID-related causes.

In health and many noninflammatory neurologic disorders, the intact blood–brain barrier prevents major central translocation by plasma immunoglobulins or cells that secrete them (*17*). Increased levels of CSF antibodies can thus result from disrupted blood–brain barrier, regulated migration of peripheral antibody-secreting cells into the CNS, or de novo antibody synthesis within the CNS. The relatively normal protein levels in patients 2 and 3 would argue against an unequivocal blood–brain barrier disruption. The lack of clear correlation between plasma and CSF titers provides some support for an active CNS process. The failure to detect CSF SARS-CoV-2 RNA does not diminish the likelihood of direct CNS infection because it is only recovered from blood in 1% of the actively infected cases (*18*), and increased levels CSF IgM are also more commonly found as evidence for CNS infection than viral recovery in other encephalitides, including those for infection with Japanese encephalitis virus (*19*), dengue virus (*20*), human parvovirus 4 (*21*), and rabies virus (*22*). At the same time, undetectable CSF RNA raises the possibility that mechanisms other than direct brain infection might account for the observed MRI and clinical changes. These changes include peri-infectious inflammation (mediated by antibodies, complement, or both) (*5*,*23*), vasculopathy, and altered neurotransmission. Until definitive neuropathologic studies or effective antiviral therapies are possible, infectious and peri-infectious etiologies need to be examined for neuro-COVID.

Increased levels of CSF multiple cytokines in these neuro-COVID patients are consistent with earlier reports of cytokine analysis of blood (*24*; M. Woodruff et al., unpub. data). We additionally identified changes shared (and not shared) by SARS-CoV-2 and HIV. Factors associated with increased levels of CSF IL-10 in patients infected with HIV should be investigated in future neuro-COVID studies, and increased levels of CSF IL-8 might uniquely provide useful information on the pathophysiology of CNS. We did not include plasma cytokine levels because their levels are much more influenced by demographic factors than their CSF counterparts (W.T. Hu et al., unpub. data). A larger cohort is necessary to better distinguish between CSF and plasma cytokine alterations, and including patients without confounding disease (e.g., SCD in patient 1) or standard MRI results can also determine the relative roles of noninfectious/inflammatory causes of encephalopathy, including hypoxia or hypercoagulability (*25*,*26*). Nevertheless, we demonstrated in these case-patients that SARS-CoV-2 antibodies are detectable in the CSF for patients with neurologic complications and are associated with selective CSF cytokine alterations. Future investigations should align neurologic outcomes with CSF infectious and immunologic profiles, such that an evidence-based treatment algorithm can be determined for preventing and treating neuro-COVID-19.
